# Overexpression of *FOXD2‐AS1* enhances proliferation and impairs differentiation of glioma stem cells by activating the NOTCH pathway via *TAF‐1*

**DOI:** 10.1111/jcmm.17268

**Published:** 2022-04-14

**Authors:** Yang Wang, Yanli Cheng, Qi Yang, Lei Kuang, Guolei Liu

**Affiliations:** ^1^ Department of Neurotumor Disease Treatment Center Taihe Hospital Hubei University of Medicine Shiyan China; ^2^ Department of Dermatology Taihe Hospital Hubei University of Medicine Shiyan China; ^3^ Department of Orthopeadic Surgery Taihe Hospital Hubei University of Medicine Shiyan China; ^4^ Department of Neurosurgery Taihe Hospital Hubei University of Medicine Shiyan China; ^5^ Department of Otorhinolaryngology South China Hospital Health Science Center Shenzhen University Shenzhen China

**Keywords:** apoptosis, differentiation, glioma, glioma stem cells, long noncoding RNA *FOXD2‐AS1*, *NOTCH1*, proliferation, stemness, *TAF‐1*

## Abstract

Emerging data have highlighted the importance of long noncoding RNAs (lncRNAs) in exerting critical biological functions and roles in different forms of brain cancer, including gliomas. In this study, we sought to investigate the role of lncRNA *FOXD2* adjacent opposite strand RNA 1 (*FOXD2*‐*AS1*) in glioma cells. First, we used sphere formation assay and flow cytometry to select U251 glioma stem cells (GSCs). Then, we quantified the expression of lncRNA *FOXD2*‐*AS1*, TATA‐box binding protein associated factor 1 (*TAF*‐*1*) and *NOTCH1* in glioma tissues and GSCs, as well as the expression of GSC stem markers, *OCT4*, *SOX2*, *Nanog*, *Nestin* and *CD133* in GSCs. Colony formation assay, sphere formation assay, and flow cytometry were used to evaluate GSC stemness. Next, the correlations among lncRNA *FOXD2*‐*AS1*, *TAF*‐*1* and *NOTCH1* were investigated. LncRNA *FOXD2*‐*AS1*, *TAF*‐*1* and *NOTCH1* were found to be elevated in glioma tissues and GSCs, and silencing lncRNA *FOXD2*‐*AS1* inhibited stemness and proliferation, while promoting apoptosis and differentiation of GSCs. LncRNA *FOXD2*‐*AS1* overexpression also led to increased *NOTCH1* by recruiting *TAF*‐*1* to the *NOTCH1* promoter region, thereby promoting stemness and proliferation, while impairing cell apoptosis and differentiation. Mechanistically, lncRNA *FOXD2*‐*AS1* elevation promoted glioma *in vivo* by activating the *NOTCH* signalling pathway via *TAF*‐*1* upregulation. Taken together, the key findings of our investigation support the proposition that downregulation of lncRNA *FOXD2*‐*AS1* presents a viable and novel molecular candidate for improving glioma treatment.

## 1 INTRODUCTION

Gliomas are the most commonly occurring brain tumours in adults, which exhibit different degrees of malignancy and histological subtypes.^1^ High‐grade gliomas are the major cause of brain tumour death.^2^ Similar to other cancers, recent evidence has demonstrated that glioma stem cells (GSCs) show tumour‐initiating properties in malignant gliomas.^3^ Radiotherapy is among the few treatments for patients with gliomas, but many gliomas become largely radio‐resistant and almost inevitably recur after treatment.^4^ Aside from radiotherapy, anti‐angiogenesis drugs against tumour cells exhibits therapeutic effects against gliomas. The anti‐angiogenesis drug known as bevacizumab is an antibody that acts against vascular endothelial growth factor, resulting in decreased malignant glioma growth.^5^ However, despite these approaches and their therapeutic effects, the treatment of malignant gliomas still remains palliative.

It is generally accepted that long noncoding RNAs (lncRNAs) can exert critical biological functions in cancers including brain tumours.^6^ LncRNAs are transcripts containing more than 200 nucleotides that are not translated into their protein products.^7^ Despite initial findings that lncRNAs play a minor physiological role despite their great abundance, an increasing amount of current research suggests an important participation in cancer biology.^8^, ^9^ Indeed, the progression and development of gliomas were found to be regulated by several lncRNAs.^10^ For example, lncRNA nuclear enriched abundant transcript 1 has been indicated to promote glioblastoma cell growth and invasion by increasing β‐catenin nuclear transport.^11^ In addition, lncRNA paternally expressed 10 has been demonstrated as a biomarker for relapse and oncologic outcomes of patients with gliomas.^12^ As revealed from the bioinformatics analysis that we conducted prior to the present investigation, lncRNA *FOXD2* adjacent opposite strand RNA 1 (*FOXD2*‐*AS1*) is a notably upregulated lncRNA in glioma samples. More importantly, the ectopic expression of lncRNA *FOXD2*‐*AS1* is indicative of poor prognosis for patients with nasopharyngeal carcinoma.^13^ The contribution of lncRNA *FOXD2*‐*AS1* to the progression of glioma has also been documented previously.^14^, ^15^ Interestingly, the NOTCH signalling pathway was reported to be regulated by lncRNA *FOXD2*‐*AS1* in colorectal cancer.^13^ The NOTCH signalling pathway is classically known for its role in determining cell fate during the development of the embryo and mature tissues.^16^ In addition, NOTCH1 signalling pathway activation can maintain the stem cell phenotype of glioma initiating cells.^17^ The inactivation of NOTCH signalling pathway was proved to be an effective target for glioma stem therapy.^18^

Furthermore, in our preliminary research *in silico*, we found a correlation between lncRNA *FOXD2*‐*AS1* and TATA‐box binding protein associated factor 1 (*TAF*‐*1*), based on the LncMAP database. *TAF*‐*1* protein is a pivotal component of the transcription factor II D complex, which plays a key role on transcription initiation.^19^ As previously reported, LINC00319 directly binds to *TAF*‐*1* and further regulates *HMGA2* in gliomas,^20^ leading us to hypothesize that lncRNA *FOXD2*‐*AS1* is involved in glioma via *TAF*‐*1* and the NOTCH signalling pathway. In our study, we aimed to explore the specific role of lncRNA *FOXD2*‐*AS1* in the proliferation and differentiation of GSCs along with the relationship between lncRNA *FOXD2*‐*AS1*/*TAF*‐*1*/*NOTCH1* signalling pathway in glioma.

## 2 MATERIALS AND METHODS

### 2.1 Microarray analysis

LncRNA microarray data GSE104267 was retrieved from Gene Expression Omnibus database. The differentially expressed lncRNAs in gliomas were screened using limma package in the Affy package of R language. The downstream transcription factors (TFs) of lncRNA *FOXD2*‐*AS1* were analysed according to LncMAP, while Gene Expression Profiling Interactive Analysis (GEPIA) was used to draw a box chart of lncRNA *FOXD2*‐*AS1* and TFs, followed by the development of overall survival curve (Group Cutoff: Quartile) regarding lncRNA *FOXD2*‐*AS1* expression based on the clinical data of patients with glioma. The target gene of TFs was analysed based on LncMAP, while KEGG pathway enrichment analysis was conducted using DAVID. The correlations of lncRNA *FOXD2*‐*AS1* with *TAF*‐*1* and of lncRNA *FOXD2*‐*AS1* with *NOTCH1* were analysed, and scatter plots were drawn.

### 2.2 Clinical sample collection

Glioma tissues were collected from 26 patients (14 males and 12 females; average age of 45.65 years, ranging from 20 to 71 years) following surgical operations in Taihe Hospital from January 2013 to May 2016. Glioma tissues were stored at −80 °C after collection. A total of 26 patients who underwent intracranial decompression in our hospital during the same period were selected as the control group with their normal brain tissues collected. After surgery, the patients received concurrent radiotherapy of temozolomide combined with adjuvant chemotherapy, which is the standard regimen for such patients (i.e. Stupp regimen). The detailed demographic information of all patients is shown in Table S1.

### 2.3 Lentiviral transduction of glioma cells

Human astrocyte cell line HA‐1800 (Catalog #1800, ScienCell, San Diego, California, USA) and glioma cell lines U251 (TCHu 58, National collection of authenticated cell cultures, Shanghai, China), LN18 (CM‐H291, Gaining Biological LTD., company, Shanghai, China), T98G (CRL‐1690, ATCC, Manassas, VA, USA), A172 (TCHu171, National collection of authenticated cell cultures, Shanghai, China) and U‐138 (HTB‐16, ATCC, Manassas, VA, USA) were selected for our experiments. The cells were incubated in high glucose Dulbecco's modified Eagle's medium (DMEM, Gibco, Carlsbad, CA, USA) containing 10% foetal bovine serum with 5% CO_2_ at 37 °C.

To isolate GSCs, U251 cells were cultured in a serum‐free medium (Hyclone, Logan, UT, USA) containing epidermal growth factor, basic fibroblast growth factor, leukaemia inhibitory factor and melanocortin 2 receptor accessory protein (final concentration of 20 ng/mL; Peprotech EC, London, UK). The suspended tumour cell spheres were then separated by immunomagnetic beads to obtain CD133 positive cells (CD133^+^ cells magnetic bead sorting Kit; Miltenyi Biotech, Bergisch Gladbach, Germany). Next, cells were prepared into single cell suspensions after the addition of immunomagnetic bead sorting solution (200 μL/10^8^ cells). Meanwhile, cells were incubated with CD133 antibody‐bead complexes (100 μL/10^5^ cells) at 4°C for 30 minutes followed by the addition of sorting solution (1 mL/10^8^ cells). Following centrifugation, the supernatant was discarded and cells were re‐suspended in sorting solution (500 μL/10^8^ cells). Cell suspension was added into separating chromatography column along with 2 mL sorting solution to elute CD133^+^ cells. CD133^+^ and CD133^−^ cells were separated and mixed with 50 μL CD133/2 (293 c3) PE antibody or 50 μL IgG2b‐PE antibody (isotype controls). Finally, cells were sorted using flow cytometry.^21^

A lentivirus‐based packaging system was designed using LV5‐GFP (lentivirus overexpression vector) and pSIH1‐H1‐copGFP (lentivirus short hairpin RNA [shRNA] fluorescent expression vector). LncRNA *FOXD2*‐*AS1* shRNA, *TAF*‐*1* shRNA and their shRNA‐negative control (sh‐NC) were constructed by GenePharma (Shanghai, China). HEK293T cells were transfected with different plasmids, and supernatants were collected after a 48‐hour culture period. Virus in the supernatants were then filtered and centrifuged, until the desired viral titre was obtained. GSCs in the logarithmic growth phase were collected and prepared into single cell suspensions containing 5 × 10^4^ cells/mL, followed by incubation in 6‐well plates with 2 mL in each well at 37 °C overnight. GSCs were then infected with lentivirus expressing short hairpin (sh)‐NC, overexpression (oe)‐NC, sh‐lncRNA *FOXD2*‐*AS1*, oe‐lncRNA *FOXD2*‐*AS1*, oe‐*TAF*‐*1*, sh‐*TAF*‐*1* or oe‐*FOXD2*‐*AS1* + sh‐*TAF*‐*1*. After 48 hours, reverse transcription quantitative polymerase chain reaction (RT‐qPCR) was used to determine expression of related genes.

### 2.4 RNA extraction and quantification

An RNA extraction kit (Qiagen, Hilden, Germany) was used to extract the total RNA from glioma tissues and GSCs. ReverTra Ace^®^ qPCR RT Master Mix with gDNA Remover Kit (Toyobo, Ohtsu, Shiga, Japan) was used to reversely‐transcribe RNA into cDNA according to the manufacturer's instructions. qPCR was then performed using Bio‐Rad CFX96 Touch™ fluorescence detection system according to instructions provided in a 2 × RealStar Green Mixture kit (GenStar BioSolutions, Beijing, China). Glyceraldehyde phosphate dehydrogenase (GAPDH) was used as internal reference. The fold change was determined using the relative quantification (2^−ΔΔCt^ method) as previously described.^22^ The primers were synthesized in Shanghai Sangon Co., Ltd. (Shanghai, China) (Table S2).

### 2.5 Western blot analysis

After 48 hours of infection, GSCs were collected and lysed using radioimmunoprecipitation (RIPA) lysis buffer (Beyotime Biotechnology, Shanghai, China). A Bicinchoninic acid kit (Pierce, USA) was used to detect the protein concentration in the supernatant. Proteins were separated by 10% sodium dodecyl sulphate polyacrylamide gel electrophoresis and transferred onto polyvinylidene fluoride membranes. The membrane was blocked with 5% skim milk and probed with rabbit antibodies against jagged canonical Notch ligand 1 (JAG1; ab7771, 1:500), NOTCH1 (ab8925, 1:500), Presenilin‐1 (PS1; ab76083, 1:5000), HES family bHLH transcription factor 1 (HES1; ab71559, 1:1000) or GAPDH (ab9485, 1:2500, internal reference). All of aforementioned antibodies were obtained from Abcam (Cambridge, UK). The membrane was then re‐probed with goat anti‐rabbit immunoglobulin G (IgG) (Santa Cruz Biotechnology Inc., Santa Cruz, CA, USA) conjugated by horseradish peroxidase (HRP) for 1 hour. Enhanced chemiluminescence (Thermo Fisher Scientific Inc., Waltham, MA, USA) was used to develop the membrane, which was visualized in a Bio‐Rad ChemiDoc™ image analysis system. Protein bands were analysed by ImageJ2x software.

### 2.6 Sphere formation assay

Single cell suspension was seeded onto ultra‐low attachment six‐well plates (Corning Inc., Corning, NY, USA) at a density of 5 × 10^3^ cells/mL and cultured in modified serum‐free DMEM. The medium was replaced every three days. After 14 days, spheres at passage 1 with a size >100 µm were counted. Then, for the secondary sphere formation, the spheres were scattered and re‐seeded in 96‐well plates at a density of approximately 100 cells per well with the number of secondary spheres calculated 10 days after incubation.

### 2.7 Immunofluorescence staining

GSCs were inoculated on polylysine‐coated slides, treated with 4% paraformaldehyde for 30 min, rinsed with PBS, treated with 0.3% Triton X‐100 at indoor temperature for 30 min, sealed with 10% goat serum, and incubated with anti‐CD133 (1:200; ab284389, Abcam), anti‐Nestin (1:200, ab18102, Abcam) and anti‐GFAP (1:200, ab7260, Abcam) overnight. Then, GSCs were incubated with the second antibody for 1 hour, and the nucleus was labelled with DAPI. Images were observed under an IX71 Olympus fluorescence microscope.

### 2.8 Dual‐luciferase reporter gene assay

Luciferase reporter plasmids were co‐transfected with oe‐NC, oe‐lncRNA *FOXD2*‐*AS1*, sh‐NC and sh‐lncRNA *FOXD2*‐*AS1*, respectively, to determine whether lncRNA *FOXD2*‐*AS1* could activate the *NOTCH1* promoter. Renilla luciferase was used as internal normalization. After a transfection period of 48 hours, cells were collected and lysed. Luciferase Assay Kit (K801‐200, BioVision, Hannover, Germany) was used to perform dual‐luciferase reporter assay in a dual fluorescent reporter analysis system (Promega, Madison, WI, USA). The activation of targeting reporter genes was determined by the ratio of relative light unit (RLU) of firefly luciferase to RLU of renilla luciferase. *NOTCH1* promoter was constructed into a luciferase reporter vector co‐transfected along with *TAF*‐*1* overexpressing plasmid into glioma cells to verify the binding relationship between TAF‐1 and *NOTCH1* promoter.

### 2.9 RNA pull‐down assay

Biotin‐labelled lncRNA *FOXD2*‐*AS1* RNA and its anti‐sense sequence were synthesized using Pierce RNA 3’desthiobiotinylation Kit (Thermo Fisher Scientific). After cells were lysed, the protein concentration (not lower than 2 mg/mL) was determined as above. A magnetic RNA‐Protein Pull‐Down kit (Thermo Fisher Scientific) was used for the subsequent procedure. A bead suspension (50 μL) was added into an Eppendorf tube, and the magnetic beads were adsorbed on the magnetic bead sorter, washed with 20 mM Tris (pH = 7.5) and then re‐suspended. After repeating the aforementioned procedure, the magnetic beads were adsorbed on the magnetic bead sorter again and re‐suspended with 100 μL of 1 × RNA Capture Buffer. The magnetic sorter was used to absorb magnetic beads, and then, the beads were washed with 20 mM Tris (pH = 7.5), followed by re‐suspension. The above procedures were repeated. A total of 100 μL 1 × Protein‐RNA‐Binding Buffer were added to the beads, followed by re‐suspension and elution. The cell lysate was incubated with 1 pmol biotin‐labelled lncRNA *FOXD2*‐*AS1* RNA and magnetic beads for 30–60 min at 4°C. Magnetic beads were absorbed and then washed with 100 μL RNA immunoprecipitation (RIP) Wash Buffer three times, followed by the addition of 50 μL Elution Buffer and incubated for 30 to 60 minutes at 4°C. LncRNA *FOXD2*‐*AS1* RNA‐binding proteins were eluted next. After determining the protein concentration, Western blot analysis was used to measure the relative protein levels.

### 2.10 RIP

The RIP was performed using an EZ‐Magna RIP RNA‐Binding Protein Immunoprecipitation kit (Millipore, Germany) according to the manufacturer's instructions to analyse the binding capacity of lncRNA *FOXD2*‐*AS1* to TAF‐1. GSCs were washed with ice‐cold PBS twice and lysed with RIPA lysis buffer, followed by 10‐min centrifugation at 14,000 rpm at 4°C. The supernatant was extracted and co‐precipitated with antibodies. Beads (50 μL) was washed, re‐suspended in 100 μL RIP Wash Buffer and incubated with rabbit antibody to *TAF*‐*1* (Abcam). The magnetic beads‐antibody complex was then re‐suspended in 900 μL RIP Wash Buffer and incubated with 100 μL cell extract at 4 °C overnight. Beads‐protein complex was collected on magnetic pedestals, and detached with protease K. The RNA was then extracted and RT‐qPCR was adopted to detect the co‐precipitated RNA.

### 2.11 Chromatin immunoprecipitation assay

GSCs were initially cultured in a 10 cm diameter dish. When the cell number reached 1 × 10^6^, the medium was discarded. The cells were crosslinked with 1% formaldehyde for 10‐min at 37°C. Cross‐linking was stopped by leaving the GSCs on ice for 5 minutes. The GSCs were collected and re‐suspended in 200 μL sodium dodecylsulphate lysis buffer, and chromatin fragmentation was performed by using ultrasound on ice. The supernatant was collected after 10‐minute cell centrifugation at 14,000 ×g at 4°C followed by dilution with ChIP buffer containing protease inhibitors. Part of the supernatant was taken as input, and the rest of the supernatant was incubated with rabbit antibody to TAF‐1 (Abcam) at 4°C overnight. Cross‐linked agar was used to precipitate the endogenous DNA‐protein complex. The supernatant was discarded after centrifugation (1000 rpm; 1 min) at 4 °C, and elution buffer was then used to elute the DNA‐protein complex. The eluted supernatant and input DNA were de‐crosslinked in a water bath for 6 hours (65°C) after addition of 5 mol/L NaCl (20 μL). The DNA fragments were then collected following detachment using protease K. RT‐qPCR was used to determine the relative levels of *NOTCH1* promoter. Primers for *NOTCH1* promoter are: Forward: CAGGGGAGACCCCCTATCC; Reverse: TGAAAGTTTTCAGAGGCCAAAAG.

### 2.12 Determination of CD133 expression in GSCs

GSCs were collected and re‐suspended using 500 μL staining buffer, followed by 5‐min centrifugation at 300 ×g and re‐suspended using 50 μL staining buffer. The cells were incubated with CD133 antibody diluent (50 mL; 1 μL anti‐CD133 [ab18235, Abcam] +49 μL staining buffer) for 30‐min at 4°C, followed by centrifugation (300 ×g). Cells were washed twice with 500 μL staining buffer and re‐suspended in 200 to 300 μL staining buffer. The expression of CD133 was analysed using a BD FACSCalibur flow cytometer.

### 2.13 Colony formation in soft agar

GSCs were collected and re‐suspended at a density of 1 × 10^6^ cells/L. Distilled water was used to prepare agarose of low solubility with a concentration between 0.7 and 1.2%. Then, 1.2% agarose was mixed with 2 × medium containing 2 × antibiotics and 20% calf serum. The 3 ml mixture was then added into an agar plate, and the bottom layer of agar was placed into an incubator. Next, 0.7% agarose was mixed with 2 × cell medium, followed by the addition of 0.2 mL cell suspension, and the mixture was then added into the agar plate. After the upper layer of agar had solidified, the plate was placed in a 37°C incubator with 5% CO_2_ for 10–14 days. Colonies containing more than 50 cells were then counted under a light microscope.

### 2.14 Flow cytometry

GSCs were seeded into 6‐well plates at 2 × 10^5^ cells/well and infected with different lentivirus. After 72 hours, GSCs were collected in 15 mL centrifuge tubes, centrifuged (800 ×g), and washed using PBS twice. GSCs were re‐suspended in 500 μL binding buffer according to the instructions provided by the AnnexinV‐FITC Apoptosis Detection Kit Ι (Becton Dickinson, Franklin Lakes, NJ, USA) and incubated with 5 μL FITC and 5 μL propidium iodide in a dark room. Flow cytometer (Becton Dickinson) was used to analyse apoptosis rates.

### 2.15 Intracranial glioma models

Serum‐free Roswell Park Memorial Institute 1640 medium (Gibco) was used to re‐suspend transfected GSCs to prepare 2 × 10^6^ cells/μL cell suspension. Fifty nude mice (aged 4–6 weeks; weighing 17–20 g) were obtained from the Shanghai SLAC Laboratory Animal Co., Ltd. (Shanghai, China). After anaesthesia with diethyl ether and routine disinfection in mice, 2 × 10^5^ cell (100 μL) GSC suspension was injected stereotaxically into the right cerebral hemisphere of the mice. On the 14th day after the injection of luciferase substrate, d‐luciferin (YEASEN, Shanghai, China), the IVIS Lumina II Imaging System (Caliper Life Sciences, Hopkinton, MA, USA) was used to capture fluorescent images. The tumour was collected and weighed at 15 days.

### 2.16 Immunohistochemistry

Four weeks following the inoculation of GSCs in nude mice, tumours were extracted, paraffin‐embedded and sectioned in 5 μm sections. The sections were then de‐waxed using xylene three times (5 min each) and rehydrated. Then, the sections were incubated with primary antibodies (Abcam) to Nestin (ab105389, 1:100), SOX2 (ab93689, 1:100), CD133 (ab226355, 1:1000) and GFAP (ab33922, 1:500) overnight at 4°C. After that, the sections were incubated with HRP‐labelled IgG (Bosterbio, Wuhan, China) for an hour. Diaminobenzidine (Bosterbio, China) was adopted to visualize the sections. The nucleus was then stained with haematoxylin (Servicebio, Wuhan, China), and the sections were dehydrated and observed under a microscope.

### 2.17 Statistical analysis

All data were processed and analysed using SPSS 21.0 statistical software (IBM Corp., Armonk, NY, USA). Measurement data were expressed as mean ± standard deviation. If the data conformed to normal distribution and homogeneity of variance, a paired *t*‐test was used to analyse tumour tissues and normal tissues. Unpaired *t*‐test was adopted to analyse differences of other two groups. Differences between multiple groups were analysed by one‐way analysis of variance (ANOVA), followed by a Tukey multiple comparisons post‐test. Comparisons among multiple groups at different time points were performed with two‐way ANOVA, and tumour volume at different time points was analysed using repeated measures ANOVA, followed by Bonferroni's *post hoc* test. Kaplan–Meier was used to analyse survival, while Log‐Rank was utilized to analyse the levels of significance in different groups. A value of *p* < 0.05 was considered statistically significant.

## 3 RESULTS

### 3.1 LncRNA *FOXD2*‐*AS1* expression is elevated in glioma tissues and GSCs

**FIGURE 1:**
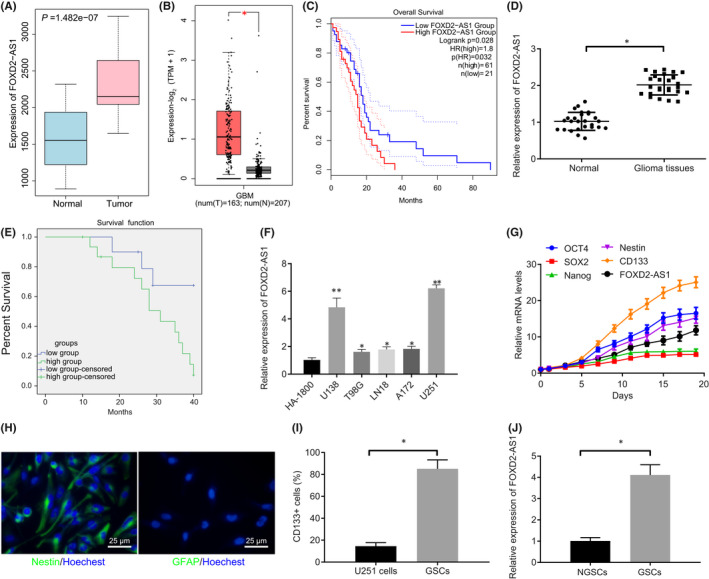
LncRNA *FOXD2*‐*AS1* was highly expressed in glioma tissues and cells as well as GSCs. A, LncRNA *FOXD2*‐*AS1* expression predicted in GSE104267 (*p* = 1.482E‐07). The box on the left represents expression of lncRNA *FOXD2*‐*AS1* in normal tissues and the box on the right represents expression in cancer tissues. B, A box plot displaying lncRNA *FOXD2*‐*AS1* expression in glioma samples (left) and normal samples (right) from TCGA database using GEPIA. C, Survival curve of patients with glioma regarding lncRNA *FOXD2*‐*AS1* expression from TCGA database using GEPIA (HR = 1.8, *p *= 0.032). D, LncRNA *FOXD2*‐*AS1* expression in glioma tissues and normal tissues detected by RT‐qPCR (n = 26). E, A Kaplan–Meier one‐way survival analysis according to lncRNA *FOXD2*‐*AS1* expression, followed by log‐rank test (n = 26). F, *FOXD2*‐*AS1* expression in glioma cell lines determined using RT‐qPCR. G, Expression of lncRNA *FOXD2*‐*AS1* and GSC markers (*OCT4*, *SOX2*, *Nanog*, *Nestin* and *CD133*) determined using RT‐qPCR. H, Expression of Nestin and GFAP in GSCs determined by immunofluorescence. I, CD133^+^ GSCs selected using flow cytometry. J, LncRNA *FOXD2*‐*AS1* expression in GSCs determined using RT‐qPCR. * *p* < 0.05. Measurement data were expressed as mean ± standard deviation. Paired *t*‐test was used to analyse comparison within group. Unpaired *t*‐test was adopted to analyse differences between two groups. Differences among multiple groups were analysed by one‐way ANOVA, followed by a Tukey multiple comparisons post‐test. Comparisons among multiple groups at different time points were performed with two‐way ANOVA. Cell experiments were conducted three times independently.

After analysis of the GSE104267 microarray dataset by R language, 101 differentially expressed lncRNAs were obtained. Among these, 19 were upregulated and 82 were downregulated. Among the upregulated lncRNAs, lncRNA *FOXD2*‐*AS1* (Figure 1A, Fig. S1) was found previously to be associated with glioma progression.^14^, ^23^, ^24^ According to GEPIA, lncRNA *FOXD2*‐*AS1* was highly expressed in gliomas from the TCGA database (Figure 1B), and predicted poor survival rate of patients (Figure 1C). Therefore, lncRNA *FOXD2*‐*AS1* was selected for the following study. To better understand the function and roles of lncRNA *FOXD2*‐*AS1* in gliomas, we first used RT‐qPCR (Figure 1D) to determine lncRNA *FOXD2*‐*AS1* expression in glioma tissues and normal tissues. We found that lncRNA *FOXD2*‐*AS1* was highly expressed in glioma tissues compared with normal tissues. Kaplan–Meier curves (Figure 1E) of patient survival revealed that patients with higher levels of lncRNA *FOXD2*‐*AS1* expression had a significantly lower survival rate compared with patients with low lncRNA *FOXD2*‐*AS1* expression. LncRNA *FOXD2*‐*AS1* expression was then analysed in glioma cell lines, identifying a distinctly higher lncRNA *FOXD2*‐*AS1* expression in glioma cell lines compared with that in the human astrocyte cell line HA‐1800. In addition, the highest lncRNA *FOXD2*‐*AS1* expression was observed in the U251 glioma cell line (Figure 1F). Thus, U251 was selected for use in our study. Taken together, we find that lncRNA *FOXD2*‐*AS1* expression was elevated in glioma tissues and cells.

The GSCs were then isolated from U251 cells, and a sphere formation assay was performed. The morphological characteristics of spherical U251 cell colonies are shown in Fig. S2A. Non‐adherent cells were collected every three days, and RT‐qPCR was used to determine expression of GSCs markers (OCT4, SOX2, Nanog, Nestin and CD133). As shown in Figure 1G, the expression of these genes gradually increased with time in culture. Immunofluorescence revealed that Nestin was positively expressed in GSCs, while almost no GFAP expression (Figure 1H). Flow cytometry was used to determine the expression of CD133 in GSCs (Figure 1I), which helped to confirm the successful isolation of GSCs. RT‐qPCR was used to determine lncRNA *FOXD2*‐*AS1* expression in GSCs, which showed a high lncRNA *FOXD2*‐*AS1* expression in GSCs (Figure 1J). These results indicated that lncRNA *FOXD2*‐*AS1* was upregulated in GSCs.

### 3.2 Silencing lncRNA *FOXD2*‐*AS1* reduces stemness and proliferation but induces apoptosis and differentiation of U251 GSCs

**FIGURE 2:**
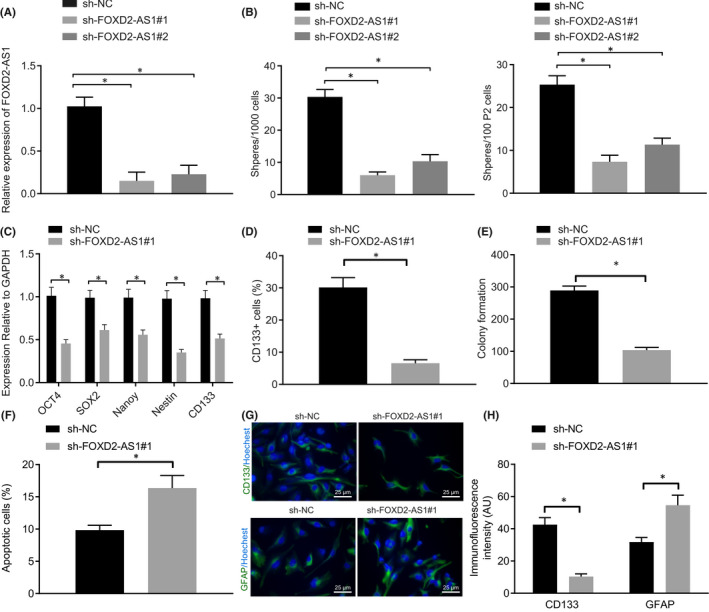
Silencing lncRNA *FOXD2*‐*AS1* suppressed stemness and proliferation but induced apoptosis and differentiation of U251 GSCs. U251 GSCs were transfected with lncRNA *FOXD2*‐*AS1* shRNAs and sh‐NC. A, Silencing efficiency of lncRNA *FOXD2*‐*AS1* shRNA in U251 GSCs determined using RT‐qPCR. B, The number of main spheres every 1000 GSCs and sphere at the second passage every 100 GSCs. C, Expression of GSC markers (*OCT4*, *SOX2*, *Nanog*, *Nestin* and *CD133*) determined using RT‐qPCR. D, CD133^+^ cells in U251 GSCs measured by flow cytometry. E, U251 GSC proliferation detected using colony formation in soft agar. F, U251 GSC apoptosis determined using flow cytometry. G‐H, GFAP and CD133 expression in GSCs determined by immunofluorescence staining (400 ×). * *p* < 0.05. Measurement data were expressed as mean ± standard deviation and analysed by unpaired *t*‐test. Cell experiments were conducted in triplicate.

To better elucidate the role of lncRNA *FOXD2*‐*AS1* in GSCs, lncRNA *FOXD2*‐*AS1* was silenced in U251 cells, and the stemness and differentiation of U251 GSCs were determined. The silencing efficiency of U251 cells treated with lncRNA *FOXD2*‐*AS1* shRNAs (sh‐lncRNA *FOXD2*‐*AS1*#1 and sh‐lncRNA *FOXD2*‐*AS1*#2) was determined through RT‐qPCR (Figure 2A). Results showed that lncRNA *FOXD2*‐*AS1* expression was significantly lower after transfection with lncRNA *FOXD2*‐*AS1* shRNAs, and that U251 cells treated with sh‐lncRNA *FOXD2*‐*AS1*#1 exhibited lower expression. Sphere formation assays demonstrated inhibition of sphere formation by U251 cells in response to sh‐*FOXD2*‐*AS1*, while sh‐*FOXD2*‐*AS1*#1 treatment exhibited a more prominent inhibitory effect on U251 sphere formation (Fig. S2B). Therefore, sh‐*FOXD2*‐*AS1*#1 was selected for subsequent functional assays. We then calculated the number of main spheres for every 1000 GSCs and the secondary sphere for every 100 GSCs, which was reduced significantly after treatment by sh‐lncRNA *FOXD2*‐*AS1* (Figure 2B). RT‐qPCR revealed that the expression of GSC stemness markers, OCT4, SOX2, Nanog, Nestin and CD133 was significantly lowered in response to sh‐lncRNA *FOXD2*‐*AS1* treatment (Figure 2C). Flow cytometry revealed an obvious reduction of CD133^+^ cells among U251 GSCs after silencing lncRNA *FOXD2*‐*AS1* (Figure 2D). Colony formation assay and flow cytometry helped to illustrate the impaired proliferation and enhanced apoptosis of U251 GSCs in the presence of sh‐lncRNA *FOXD2*‐*AS1* (Figure 2E,F). Immunofluorescence staining was employed to determine the expression of astrocyte marker GFAP and stem cell marker CD133 in U251 GSCs. As depicted in Figure 2G,H, GFAP expression was significantly upregulated, while CD133 expression was downregulated in response to sh‐lncRNA *FOXD2*‐*AS1*. These findings suggested that silencing lncRNA *FOXD2*‐*AS1* reduced stemness and proliferation, but promoted apoptosis and differentiation of U251 GSCs.

### 3.3 LncRNA *FOXD2*‐*AS1* elevates *NOTCH1* by recruiting *TAF*‐*1* to promoter region of *NOTCH1* in GSCs

**FIGURE 3:**
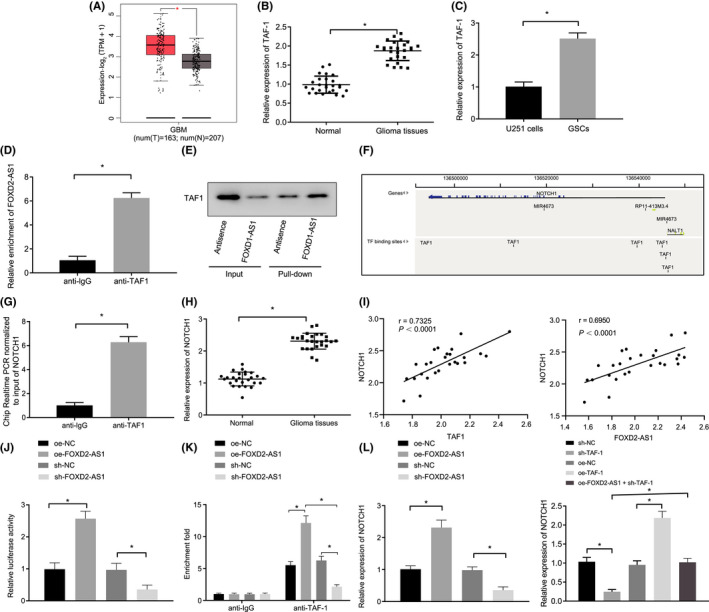
LncRNA *FOXD2*‐*AS1* upregulation elevates *NOTCH1* expression by recruiting *TAF*‐*1* to the *NOTCH1* promoter region in GSCs. A, *TAF*‐*1* expression in glioma tissues predicted in GEPIA website. B, *TAF*‐*1* expression in glioma tissues and normal tissues determined by RT‐qPCR (n = 26). C, *TAF*‐*1* expression in U251 GSCs assessed using RT‐qPCR and Western blot analysis. D, The binding relationship between lncRNA *FOXD2*‐*AS1* and *TAF*‐*1* in U251 GSCs verified using RIP normalized to IgG. E, The binding relationship between lncRNA *FOXD2*‐*AS1* and *TAF*‐*1* in U251 GSCs verified using RNA pull‐down. F, Binding sites between *TAF*‐*1* and *NOTCH1* predicted by GTRD website. G, Binding between TAF‐1 and *NOTCH1* promoter determined by ChIP assay. H, *NOTCH1* expression in glioma tissues and normal tissues assessed by RT‐qPCR and Western blot analysis (n = 26). I, Pearson's correlation analysis of relationship between lncRNA *FOXD2*‐*AS1* and *NOTCH1* expression (*p* < 0.0001, R = 0.6950), as well as between *TAF*‐*1* and *NOTCH1* expression (*p* < 0.0001, R = 0.7325). J, Targeting relationship between lncRNA *FOXD2*‐*AS1* and *NOTCH1* promoter detected by dual‐luciferase reporter gene assay. K, Binding of *TAF*‐*1* and *NOTCH1* after alteration of lncRNA *FOXD2*‐*AS1* evaluated by ChIP assay. L, *NOTCH1* expression in response to alteration of lncRNA *FOXD2*‐*AS1* and silencing or overexpressed *TAF*‐*1* assessed by RT‐qPCR and Western blot analysis. * *p* < 0.05. Measurement data were expressed as mean ±standard deviation. Paired *t*‐test was used to analyse tumour tissues and normal tissues. Unpaired *t*‐test was adopted to analyse differences of other two groups. Differences among multiple groups were analysed by one‐way ANOVA, followed by a Tukey multiple comparisons post‐test. Pearson's correlation analysis was used. Cell experiments were repeated in triplicate.

The interaction of lncRNA *FOXD2*‐*AS1* and *TAF*‐*1* with *NOTCH1* was found based on the LncMAP database, and *TAF*‐*1* was initially predicted to be highly expressed in glioma according to the GEPIA website (Figure 3A). Moreover, the activation of NOTCH signalling pathway was previously found to be associated with GSC differentiation and metastasis.^17^, ^25^, ^26^ We therefore hypothesized that lncRNA *FOXD2*‐*AS1* could potentially activate the NOTCH signalling pathway by regulating *NOTCH1* via *TAF*‐*1*. To test this hypothesis, RT‐qPCR was initially adopted to determine *TAF*‐*1* expression in glioma tissues. *TAF*‐*1* expression was found to be higher in glioma tissues than in normal tissues (Figure 3B). Moreover, RT‐qPCR and Western blot analysis revealed that the level of *TAF*‐*1* was higher in U251 GSCs (Figure 3C and Fig. S3A). As shown in Figure 3D,E, lncRNA *FOXD2*‐*AS1* bound to *TAF*‐*1* in U251 GSCs, which was further verified using RIP and RNA pull‐down assays. We initially predicated that multiple binding sites existed between *TAF*‐*1* and *NOTCH1* (Figure 3F) according to the GTRD website. A ChIP assay revealed that TAF‐1 could indeed bind to *NOTCH1* at its promoter region (Figure 3G). RT‐qPCR and Western blot analysis depicted that *NOTCH1* expression in glioma tissues was much higher than that in normal tissues (Figure 3H and Fig. S3B). Pearson's correlation analysis also found a positive relationship between the expression of lncRNA *FOXD2*‐*AS1* and *NOTCH1*, as well as that between the expression of *TAF*‐*1* and *NOTCH1* (Figure 3I). Dual‐luciferase reporter gene assays were carried out to help verify the relationship between lncRNA *FOXD2*‐*AS1* and *NOTCH1* promoter. The results revealed that the *NOTCH1* promoter was activated in response to oe‐lncRNA *FOXD2*‐*AS1* treatment, while the opposite occurred in response to sh‐lncRNA *FOXD2*‐*AS1*. This strongly suggested that lncRNA *FOXD2*‐*AS1* could specifically activate *NOTCH1* (Figure 3J). ChIP assay (Figure 3K) showed that the enrichment of *TAF*‐*1* in *NOTCH1* promoter was inhibited in response to sh‐lncRNA *FOXD2*‐*AS1*, while the opposite effect was observed after treatment with oe‐lncRNA *FOXD2*‐*AS1*. RT‐qPCR and Western blot analysis (Figure 3L and Fig. S3C, D) showed that *NOTCH1* expression was upregulated upon treatment with oe‐lncRNA *FOXD2*‐*AS1*, but was downregulated in response to sh‐lncRNA *FOXD2*‐*AS1*. Silencing *TAF*‐*1* reduced *NOTCH1* expression, but was elevated in response to oe‐*TAF*‐*1*; the reduced *NOTCH1* expression was later restored in GSCs treated with oe‐lncRNA *FOXD2*‐*AS1* + sh‐*TAF*‐*1*. Taken together, the overexpression of lncRNA *FOXD2*‐*AS1* upregulated *NOTCH1* by recruiting *TAF*‐*1* to *NOTCH1* promoter region in GSCs.

### 3.4 Overexpression of lncRNA *FOXD2*‐*AS1* activates the NOTCH signalling pathway by recruiting *TAF*‐*1*, thereby promoting GSC stemness and repression of GSC differentiation

**FIGURE 4:**
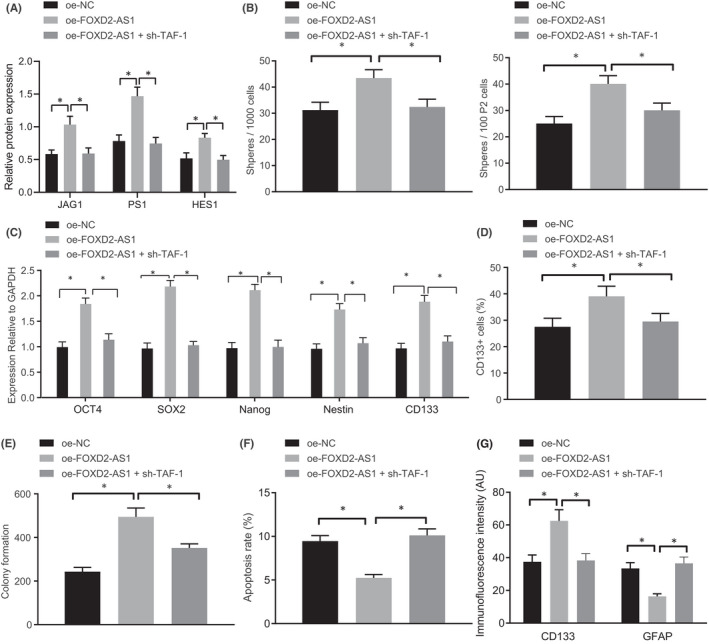
LncRNA *FOXD2*‐*AS1* elevation induced GSC stemness and inhibited GSC differentiation via activation of the NOTCH signalling pathway via *TAF*‐*1* recruitment. GSCs were transfected with oe‐NC, oe‐lncRNA *FOXD2*‐*AS1* or oe‐lncRNA *FOXD2*‐*AS1* + sh‐*TAF*‐*1*. A, Western blot analysis of expression of NOTCH pathway related genes (JAG1, PS1 and HES1). B, The number of main spheres every 1000 GSCs and sphere at the second passage every 100 GSCs. C, *OCT4*, *SOX2*, *Nanog*, *Nestin* and *CD133* expression detected using RT‐qPCR. D, CD133^+^ cells in U251 GSCs assessed by flow cytometry. E, U251 GSC proliferation measured by colony formation in soft agar. F, U251 GSC apoptosis evaluated by flow cytometry. G, GFAP and CD133 expression in GSCs assessed by immunofluorescence staining. * *p* < 0.05. Measurement data were expressed as mean ± standard deviation and analysed by one‐way ANOVA, followed by a Tukey multiple comparisons post‐test. Cell experiments were repeated in triplicate.

Since lncRNA *FOXD2*‐*AS1* was able to upregulate *NOTCH1*, we decided to explore whether the entire NOTCH pathway could be similarly activated. We first conducted a Western blot analysis to determine expression of NOTCH pathway related genes (*JAG1*, *PS1* and *HES1*). As described in Figure 4A, JAG1, PS1 and HES1 expression in GSCs were significantly elevated in response to oe‐lncRNA *FOXD2*‐*AS1*, which was reversed by silencing *TAF*‐*1*.

It was previously reported that *NOTCH1* was involved in regulating the stemness of GSCs.^27^, ^28^, ^29^ We thus hypothesized that the lncRNA *FOXD2*‐*AS1*‐*TAF*‐*1*‐*NOTCH1* axis might be associated with stemness and differentiation of GSCs. Sphere formation assays revealed a drastically enhanced sphere forming ability of U251 GSCs in the presence of oe‐lncRNA *FOXD2*‐*AS1* (Fig. S2C), which could be reversed when treated with oe‐lncRNA *FOXD2*‐*AS1* + sh‐*TAF*‐*1*. The number of main spheres in every 1000 GSCs and the secondary spheres of every 100 GSCs was increased in response to oe‐lncRNA *FOXD2*‐*AS1*, which was restored following treatment of oe‐lncRNA *FOXD2*‐*AS1* + sh‐*TAF*‐*1* (Figure 4B). *OCT4*, *SOX2*, *Nanog*, *Nestin* and *CD133* expression in GSCs assessed by RT‐qPCR demonstrated that the expression of these markers was significantly enhanced after overexpression of lncRNA *FOXD2*‐*AS1*, but that these effects were abrogated by treatment with oe‐lncRNA *FOXD2*‐*AS1* + sh‐*TAF*‐*1* (Figure 4C). Flow cytometry (Figure 4D) revealed an obvious increase in the number of CD133^+^ cells in U251 GSCs in response to oe‐lncRNA *FOXD2*‐*AS1* treatment while this effect was rescued after transfection with oe‐lncRNA *FOXD2*‐*AS1* + sh‐*TAF*‐*1*. Colony formation in soft agar and flow cytometry demonstrated enhanced proliferation and impaired apoptosis of GSCs in response to oe‐lncRNA *FOXD2*‐*AS1*, which was abolished by treatment with oe‐lncRNA *FOXD2*‐*AS1* + sh‐*TAF*‐*1* (Figure 4E,F). Immunofluorescence staining indicated that GFAP expression was significantly lowered, while CD133 expression was increased in response to oe‐lncRNA *FOXD2*‐*AS1*. This effect was rescued after transfection with oe‐lncRNA *FOXD2*‐*AS1* + sh‐*TAF*‐*1* (Figure 4G). Therefore, the overexpression of lncRNA *FOXD2*‐*AS1* upregulated *NOTCH1* by recruiting *TAF*‐*1*, thereby activating the NOTCH signalling pathway to promote GSC stemness and repress GSC differentiation.

### 3.5 Silencing lncRNA *FOXD2*‐*AS1* suppresses the NOTCH signalling pathway and glioma development *in vivo* by inhibiting *TAF*‐*1*

**FIGURE 5:**
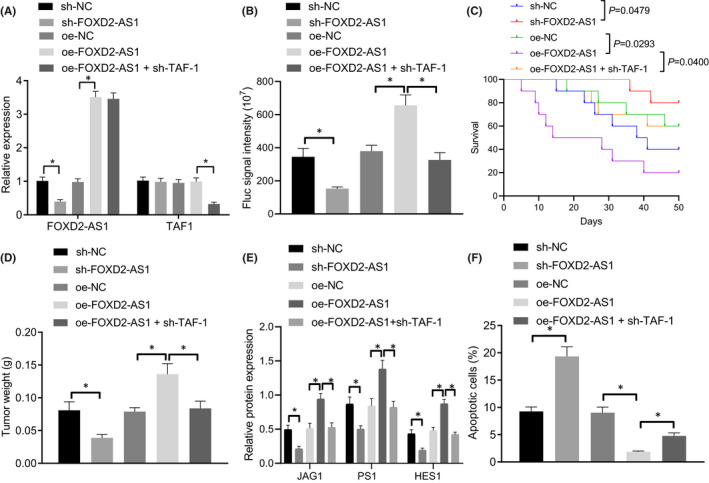
Glioma development and NOTCH signalling pathway *in vivo* were suppressed after silencing of lncRNA *FOXD2*‐*AS1* via *TAF*‐*1*. Nude mice were injected with GSCs expressing sh‐NC, sh‐lncRNA *FOXD2*‐*AS1*, oe‐NC, oe‐lncRNA *FOXD2*‐*AS1* or oe‐lncRNA *FOXD2*‐*AS1* + sh‐*TAF*‐*1*. A, LncRNA *FOXD2*‐*AS1* and *TAF*‐*1* expression in nude mice determined by RT‐qPCR. B, Tumour growth observed through the IVIS Lumina II Imaging System, n = 10. C, Survival of mice after different treatment. D, Tumour weight recorded on 15 days, n = 10. E, Western blot analysis of JAG1, PS1 and HES1 expression in mice. F, GSC apoptosis in nude mice determined using flow cytometry. * *p* < 0.05. Measurement data were expressed as mean ± standard deviation. Differences among multiple groups were analysed by one‐way ANOVA, followed by a Tukey multiple comparisons post‐test. Tumour volume among multiple groups at different time points was analysed using repeated measures ANOVA. Cell experiments were repeated in triplicate.

To better understand the role of the lncRNA *FOXD2*‐*AS1*‐*TAF*‐*1*‐*NOTCH1* axis in glioma, we established tumour xenografts in nude mice. Nude mice were injected with lncRNA altered *FOXD2*‐*AS1* and silenced *TAF*‐*1* GSCs, and RT‐qPCR was then used to determine lncRNA *FOXD2*‐*AS1* and *TAF*‐*1* expression levels in tumours from the mice. Results showed that lncRNA *FOXD2*‐*AS1* expression was reduced and *TAF*‐*1* expression remained unchanged when mice were injected with GSCs expressing sh‐lncRNA *FOXD2*‐*AS1*. In addition, we observed an increase in lncRNA *FOXD2*‐*AS1* and unchanged *TAF*‐*1* expression levels after injection with overexpressed lncRNA *FOXD2*‐*AS1* GSCs. However, after injection with GSCs stably expressing lncRNA *FOXD2*‐*AS1* + sh‐*TAF*‐*1*, lncRNA *FOXD2*‐*AS1* upregulation and TAF‐1 downregulation were observed (Figure 5A). Tumour growth and weight were also found to be markedly reduced and survival was prolonged in response to sh‐lncRNA *FOXD2*‐*AS1*. However, lncRNA *FOXD2*‐*AS1* overexpression elevated tumour growth and weight as well as shortened survival, which was blocked by oe‐lncRNA *FOXD2*‐*AS1* + sh‐*TAF*‐*1* (Fig. S4A, Figure 5B‐D). Western blot analysis demonstrated inhibited expression of JAG1, PS1 and HES1 expression in response to sh‐lncRNA *FOXD2*‐*AS1*, while increased expression of JAG1, PS1 and HES1 was identified in the presence of oe‐lncRNA *FOXD2*‐*AS1*. The effect of oe‐lncRNA *FOXD2*‐*AS1* on the expression of *JAG1*, *PS1* and *HES1* was restored by oe‐lncRNA *FOXD2*‐*AS1* + sh‐*TAF*‐*1* (Figure 5E and Fig. S3E). Flow cytometry helped to demonstrate that GSC apoptosis was enhanced in response to sh‐lncRNA *FOXD2*‐*AS1*. On the contrary, GSC apoptosis was decreased in response to oe‐lncRNA *FOXD2*‐*AS1*, and subsequently restored by oe‐lncRNA *FOXD2*‐*AS1* + sh‐*TAF*‐*1* (Figure 5F). Immunohistochemistry revealed that *NOTCH1*, *Nestin*, *SOX2* and *CD133* expression were drastically reduced and GFAP expression was elevated in response to sh‐lncRNA *FOXD2*‐*AS1*. However, the overexpression of lncRNA *FOXD2*‐*AS1* led to increased *NOTCH1*, *Nestin*, *SOX2* and *CD133* expression but decreased *GFAP* expression in mice, which was abrogated by oe‐lncRNA *FOXD2*‐*AS1* + sh‐*TAF*‐*1* (Fig. S4B). In conclusion, lncRNA *FOXD2*‐*AS1*‐*TAF*‐*1*‐*NOTCH1* axis promoted glioma *in vivo*.

## 4 DISCUSSION

Gliomas are categorized into five principal groups based on tumour markers and into different subgroups according to their underlying pathogenesis.^30^ The manifest diversity of different types of gliomas poses a pressing challenge in their treatment. Malignant gliomas constitute as the most aggressive subtype of gliomas. There remains a lack of effective therapeutic treatment regimen, which is due in part to the unhampering proliferation of cancer stem cells, which is a known aetiology of malignant gliomas.^31^ Currently, the specific molecular mechanism underlying GSS proliferation and differentiation remains unclear. In the current study, we found that the overexpression of lncRNA *FOXD2*‐*AS1* upregulated *NOTCH1* by recruiting *TAF*‐*1* to the *NOTCH1* promoter region. Moreover, the overexpression of lncRNA *FOXD2*‐*AS1* led to induced proliferation, and reduced differentiation and apoptosis of U251 GSCs, thereby promoting the progression of glioma both *in vivo* and *in vitro*.

The first main finding of this study was that lncRNA *FOXD2*‐*AS1* expression was potently elevated in GSCs and glioma tissues. A recent study demonstrated that lncRNA *FOXD2*‐*AS1* upregulation resulted in larger tumour sizes, an extensive invasion depth, distant metastasis and advanced TNM stage in cancer.^32^ Concordant with our study, a prior study revealed that lncRNA *FOXD2*‐*AS1* was overexpressed in glioma tissues and cells.^23^ To better understand the specific mechanism underlying the regulatory role of lncRNA *FOXD2*‐*AS1* in glioma cells, we performed a series of assays demonstrating that lncRNA *FOXD2*‐*AS1* upregulation led to elevated *NOTCH1* expression by recruiting *TAF*‐*1* to the *NOTCH1* promoter region. Similarly, a prior study revealed that lncRNA TRERNA1 was able to recruit EHMT2 onto the CDH1 promoter region to promote hepatocellular carcinoma development.^33^ Moreover, transcription factors such as PolII were found to be recruited to the *NOTCH1* promoter.^34^

In our study, we also found that lncRNA *FOXD2*‐*AS1* overexpression was able to induce GSC stemness and proliferation as well as inhibiting GSC differentiation and apoptosis through activation of the NOTCH signalling pathway by recruiting *TAF*‐*1*, as evidenced by increased expression of *OCT4*, *SOX2*, *Nanog*, *Nestin* and *CD133*. Previous work showed that the expression of *OCT4*, *SOX2* and *Nanog* correlated positively with tumour malignancy in human gliomas.^35^ In addition, a previous study also noted that CD133 and Nestin were cancer cell stemness markers, whereby CD133 and Nestin overexpression was correlated with worse prognosis in terms of overall survival.^36^ A recent study found that lncRNA *FOXD2*‐*AS1* upregulation resulted in larger tumour size, greater invasion depth, distant metastasis and advanced tumour node metastasis staging in cancer.^37^ Moreover, the knockdown of lncRNA *FOXD2*‐*AS1* significantly suppressed proliferation, and promoted apoptosis of glioma cells.^23^ The repression of lncRNA *FOXD2*‐*AS1* was reported to reduce proliferation, migration, invasion, stemness of glioma cells and impaired tumour growth in transplanted tumours,^24^ which was consistent to what was found in our study. *TAF*‐*1* was previously found to be functional in glioma targeted by lncRNA LINC00319.^20^ We found that *NOTCH1* was expressed at higher levels in GSCs and glioma tissues, consistent with results in a prior study.^38^ The *NOTCH* signalling pathway plays a pivotal role on the self‐renewal, proliferation and differentiation of neural stem cells or neural precursor cells.^39^ In addition, *NOTCH1* downregulation exhibits inhibitory effects on GSC proliferation,^29^ which has been regarded to present an effective strategy for GSC‐targeting therapy.^18^ Therefore, in our study, we demonstrated that lncRNA *FOXD2*‐*AS1* could activate the NOTCH signalling pathway. Likewise, lncRNA *FOXD2*‐*AS1* knockdown inactivated NOTCH signalling pathway to suppress colorectal cancer development.^40^

## 5 CONCLUSION

**FIGURE 6:**
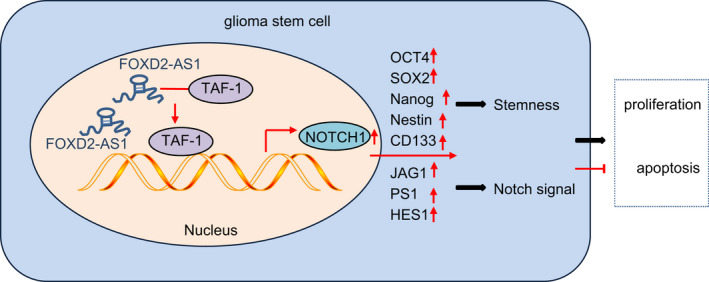
Increased lncRNA *FOXD2*‐*AS1* upregulates *NOTCH1* by recruiting *TAF*‐*1* to the *NOTCH1* promoter region, thereby promoting GSC stemness and proliferation as well as repressing GSC differentiation and apoptosis.

This study provides new insights into the molecular mechanism of how lncRNA *FOXD2*‐*AS1* is involved in the pathogenesis of gliomas. LncRNA *FOXD2*‐*AS1* is upregulated in GSCs and glioma tissues, which helps to positively regulate *NOTCH1* expression. Increased levels of the lncRNA *FOXD2*‐*AS1* also upregulate *NOTCH1* by recruiting *TAF*‐*1* to the *NOTCH1* promoter region, thereby promoting GSC stemness and proliferation as well as repressing GSC differentiation and apoptosis (Figure 6). The mechanism elucidated in our investigation may present new therapeutic targets for treating gliomas.

## CONSENT FOR PUBLICATION

Not applicable.

## CONFLICTS OF INTEREST

The authors declared that there are no conflicts of interest.

## AUTHOR CONTRIBUTIONS

**Yang Wang:** Writing – review & editing (equal). **Yanli Cheng:** Data curation (equal); Formal analysis (equal). **Qi Yang:** Writing – original draft (equal); Writing – review & editing (equal). **Lei Kuang:** Data curation (equal); Formal analysis (equal). **Guolei Liu:** Writing – original draft (equal).

## Supporting information

Fig S1

Fig S2

Fig S3

Fig S4

Table S1‐S2

## Data Availability

The data that support the findings of this study are available from the corresponding author upon reasonable request.

## References

[1] Zhang X , Sun S , Pu JK , et al. Long non‐coding RNA expression profiles predict clinical phenotypes in glioma. Neurobiol Dis. 2012;48:1‐8. 22709987 10.1016/j.nbd.2012.06.004

[2] Venkatesh HS , Johung TB , Caretti V , et al. Neuronal activity promotes glioma growth through neuroligin‐3 secretion. Cell. 2015;161:803‐816. 25913192 10.1016/j.cell.2015.04.012 PMC4447122

[3] Ma Q , Long W , Xing C , et al. Cancer stem cells and immunosuppressive microenvironment in glioma. Front Immunol. 2018;9:2924. 30619286 10.3389/fimmu.2018.02924 PMC6308128

[4] Wang J , Wakeman TP , Lathia JD , et al. Notch promotes radioresistance of glioma stem cells. Stem Cells. 2010;28:17‐28. 19921751 10.1002/stem.261 PMC2825687

[5] Wu HB , Yang S , Weng HY , et al. Autophagy‐induced KDR/VEGFR‐2 activation promotes the formation of vasculogenic mimicry by glioma stem cells. Autophagy. 2017;13:1528‐1542. 28812437 10.1080/15548627.2017.1336277 PMC5612353

[6] Ramos AD , Attenello FJ , Lim DA . Uncovering the roles of long noncoding RNAs in neural development and glioma progression. Neurosci Lett. 2016;625:70‐79. 26733304 10.1016/j.neulet.2015.12.025 PMC5094911

[7] Perkel JM . Visiting "noncodarnia". Biotechniques. 2013;54(301):303‐304. 10.2144/000114037 23750541

[8] Mercer TR , Dinger ME , Mattick JS . Long non‐coding RNAs: insights into functions. Nat Rev Genet. 2009;10:155‐159. 19188922 10.1038/nrg2521

[9] Dinger ME , Amaral PP , Mercer TR , Mattick JS . Pervasive transcription of the eukaryotic genome: functional indices and conceptual implications. Brief Funct Genomic Proteomic. 2009;8:407‐423. 19770204 10.1093/bfgp/elp038

[10] Chen Y , Wu JJ , Lin XB , et al. Differential lncRNA expression profiles in recurrent gliomas compared with primary gliomas identified by microarray analysis. Int J Clin Exp Med. 2015;8:5033‐5043. 26131076 PMC4483865

[11] Chen Q , Cai J , Wang Q , et al. Long noncoding RNA NEAT1, regulated by the EGFR pathway, contributes to glioblastoma progression through the WNT/beta‐catenin pathway by scaffolding EZH2. Clin Cancer Res. 2018;24:684‐695. 29138341 10.1158/1078-0432.CCR-17-0605

[12] Xiao H , Ding N , Liao H , et al. Prediction of relapse and prognosis by expression levels of long noncoding RNA PEG10 in glioma patients. Medicine (Baltimore). 2019;98:e17583. 31702614 10.1097/MD.0000000000017583 PMC6855493

[13] Chen G , Sun W , Hua X , Zeng W , Yang L . Long non‐coding RNA FOXD2‐AS1 aggravates nasopharyngeal carcinoma carcinogenesis by modulating miR‐363‐5p/S100A1 pathway. Gene. 2018;645:76‐84. 29248577 10.1016/j.gene.2017.12.026

[14] Ni W , Xia Y , Bi Y , Wen F , Hu D , Luo L . FoxD2‐AS1 promotes glioma progression by regulating miR‐185‐5P/HMGA2 axis and PI3K/AKT signaling pathway. Aging (Albany NY). 2019;11:1427‐1439. 30860979 10.18632/aging.101843 PMC6428107

[15] Yu DJ , Li YH , Zhong M . MicroRNA‐597 inhibits NSCLC progression through negatively regulating CDK2 expression. Eur Rev Med Pharmacol Sci. 2020;24:4288‐4297. 32373965 10.26355/eurrev_202004_21009

[16] Capaccione KM , Pine SR . The Notch signaling pathway as a mediator of tumor survival. Carcinogenesis. 2013;34:1420‐1430. 23585460 10.1093/carcin/bgt127 PMC3697894

[17] Zhang C , Hai L , Zhu M , et al. Actin cytoskeleton regulator Arp2/3 complex is required for DLL1 activating Notch1 signaling to maintain the stem cell phenotype of glioma initiating cells. Oncotarget. 2017;8:33353‐33364. 28380416 10.18632/oncotarget.16495 PMC5464873

[18] Wang J , Yan Z , Liu X , Che S , Wang C , Yao W . Alpinetin targets glioma stem cells by suppressing Notch pathway. Tumour Biol. 2016;37:9243‐9248. 26768745 10.1007/s13277-016-4827-2

[19] Gudmundsson S , Wilbe M , Filipek‐Gorniok B , et al. TAF1, associated with intellectual disability in humans, is essential for embryogenesis and regulates neurodevelopmental processes in zebrafish. Sci Rep. 2019;9:10730. 31341187 10.1038/s41598-019-46632-8 PMC6656882

[20] Li Q , Wu Q , Li Z , et al. LncRNA LINC00319 is associated with tumorigenesis and poor prognosis in glioma. Eur J Pharmacol. 2019;861:172556. 10.1016/j.ejphar.2019.172556 31325436

[21] Wang ZF , Liao F , Wu H , Dai J . Glioma stem cells‐derived exosomal miR‐26a promotes angiogenesis of microvessel endothelial cells in glioma. J Exp Clin Cancer Res. 2019;38:201. 31101062 10.1186/s13046-019-1181-4 PMC6525364

[22] Ayuk SM , Abrahamse H , Houreld NN . The role of photobiomodulation on gene expression of cell adhesion molecules in diabetic wounded fibroblasts in vitro. J Photochem Photobiol B. 2016;161:368‐374. 27295416 10.1016/j.jphotobiol.2016.05.027

[23] Dong H , Cao W , Xue J . Long noncoding FOXD2‐AS1 is activated by CREB1 and promotes cell proliferation and metastasis in glioma by sponging miR‐185 through targeting AKT1. Biochem Biophys Res Commun. 2019;508:1074‐1081. 30553445 10.1016/j.bbrc.2018.12.050

[24] Shen F , Chang H , Gao G , Zhang B , Li X , Jin B . Long noncoding RNA FOXD2‐AS1 promotes glioma malignancy and tumorigenesis via targeting miR‐185‐5p/CCND2 axis. J Cell Biochem. 2019;120:9324‐9336. 30520141 10.1002/jcb.28208

[25] Li Y , He ZC , Zhang XN , et al. Stanniocalcin‐1 augments stem‐like traits of glioblastoma cells through binding and activating NOTCH1. Cancer Lett. 2018;416:66‐74. 29196129 10.1016/j.canlet.2017.11.033

[26] Cenciarelli C , Marei HE , Zonfrillo M , et al. The interference of Notch1 target Hes1 affects cell growth, differentiation and invasiveness of glioblastoma stem cells through modulation of multiple oncogenic targets. Oncotarget. 2017;8:17873‐17886. 28157712 10.18632/oncotarget.15013 PMC5392293

[27] Zeng F , Chen H , Zhang Z , et al. Regulating glioma stem cells by hypoxia through the Notch1 and Oct3/4 signaling pathway. Oncol Lett. 2018;16:6315‐6322. 30405767 10.3892/ol.2018.9442 PMC6202516

[28] Cui C , Chen X , Liu Y , et al. beta1,4‐Galactosyltransferase V activates Notch1 signaling in glioma stem‐like cells and promotes their transdifferentiation into endothelial cells. J Biol Chem. 2018;293:2219‐2230. 29269413 10.1074/jbc.RA117.000682 PMC5808780

[29] Feng HB , Wang J , Jiang HR , et al. beta‐Elemene selectively inhibits the proliferation of glioma stem‐like cells through the downregulation of Notch1. Stem Cells Transl Med. 2017;6:830‐839. 28297578 10.5966/sctm.2016-0009 PMC5442766

[30] Eckel‐Passow JE , Lachance DH , Molinaro AM , et al. Glioma groups based on 1p/19q, IDH, and TERT promoter mutations in tumors. N Engl J Med. 2015;372:2499‐2508. 26061753 10.1056/NEJMoa1407279 PMC4489704

[31] Eyler CE , Wu Q , Yan K , et al. Glioma stem cell proliferation and tumor growth are promoted by nitric oxide synthase‐2. Cell. 2011;146:53‐66. 21729780 10.1016/j.cell.2011.06.006 PMC3144745

[32] Han X , Tang Y , Dai Y , et al. MiR‐889 promotes cell growth in human non‐small cell lung cancer by regulating KLF9. Gene. 2019;699:94‐101. 30849540 10.1016/j.gene.2019.02.077

[33] Song W , Gu Y , Lu S , et al. LncRNA TRERNA1 facilitates hepatocellular carcinoma metastasis by dimethylating H3K9 in the CDH1 promoter region via the recruitment of the EHMT2/SNAI1 complex. Cell Prolif. 2019;52:e12621. 31012192 10.1111/cpr.12621 PMC6668973

[34] Lambertini C , Pantano S , Dotto GP . Differential control of Notch1 gene transcription by Klf4 and Sp3 transcription factors in normal versus cancer‐derived keratinocytes. PLoS One. 2010;5:e10369. 20442780 10.1371/journal.pone.0010369 PMC2860992

[35] Guo Y , Liu S , Wang P , et al. Expression profile of embryonic stem cell‐associated genes Oct4, Sox2 and Nanog in human gliomas. Histopathology. 2011;59:763‐775. 22014056 10.1111/j.1365-2559.2011.03993.x

[36] Wu B , Sun C , Feng F , Ge M , Xia L . Do relevant markers of cancer stem cells CD133 and Nestin indicate a poor prognosis in glioma patients? A systematic review and meta‐analysis. J Exp Clin Cancer Res. 2015;34:44. 25967234 10.1186/s13046-015-0163-4 PMC4436020

[37] Zhou L , Li Z , Shao X , et al. Prognostic value of long non‐coding RNA FOXD2‐AS1 expression in patients with solid tumors. Pathol Res Pract. 2019;215:152449. 10.1016/j.prp.2019.152449 31378453

[38] Jiang L , Wu J , Chen Q , Hu X , Li W , Hu G . Notch1 expression is upregulated in glioma and is associated with tumor progression. J Clin Neurosci. 2011;18:387‐390. 21251836 10.1016/j.jocn.2010.07.131

[39] Liu J , Sato C , Cerletti M , Wagers A . Notch signaling in the regulation of stem cell self‐renewal and differentiation. Curr Top Dev Biol. 2010;92:367‐409. 20816402 10.1016/S0070-2153(10)92012-7

[40] Yang X , Duan B , Zhou X . Long non‐coding RNA FOXD2‐AS1 functions as a tumor promoter in colorectal cancer by regulating EMT and Notch signaling pathway. Eur Rev Med Pharmacol Sci. 2017;21:3586‐3591. 28925486

