# Supplementary figures and images for: Overexpression of FOXD2‐AS1 enhances proliferation and impairs differentiation of glioma stem cells by activating the NOTCH pathway via TAF‐1
Source: J Cell Mol Med. 2022 Apr 14;26(9):2620–32. doi: 10.1111/jcmm.17268 (PMC9077300; doi:10.1111/jcmm.17268)

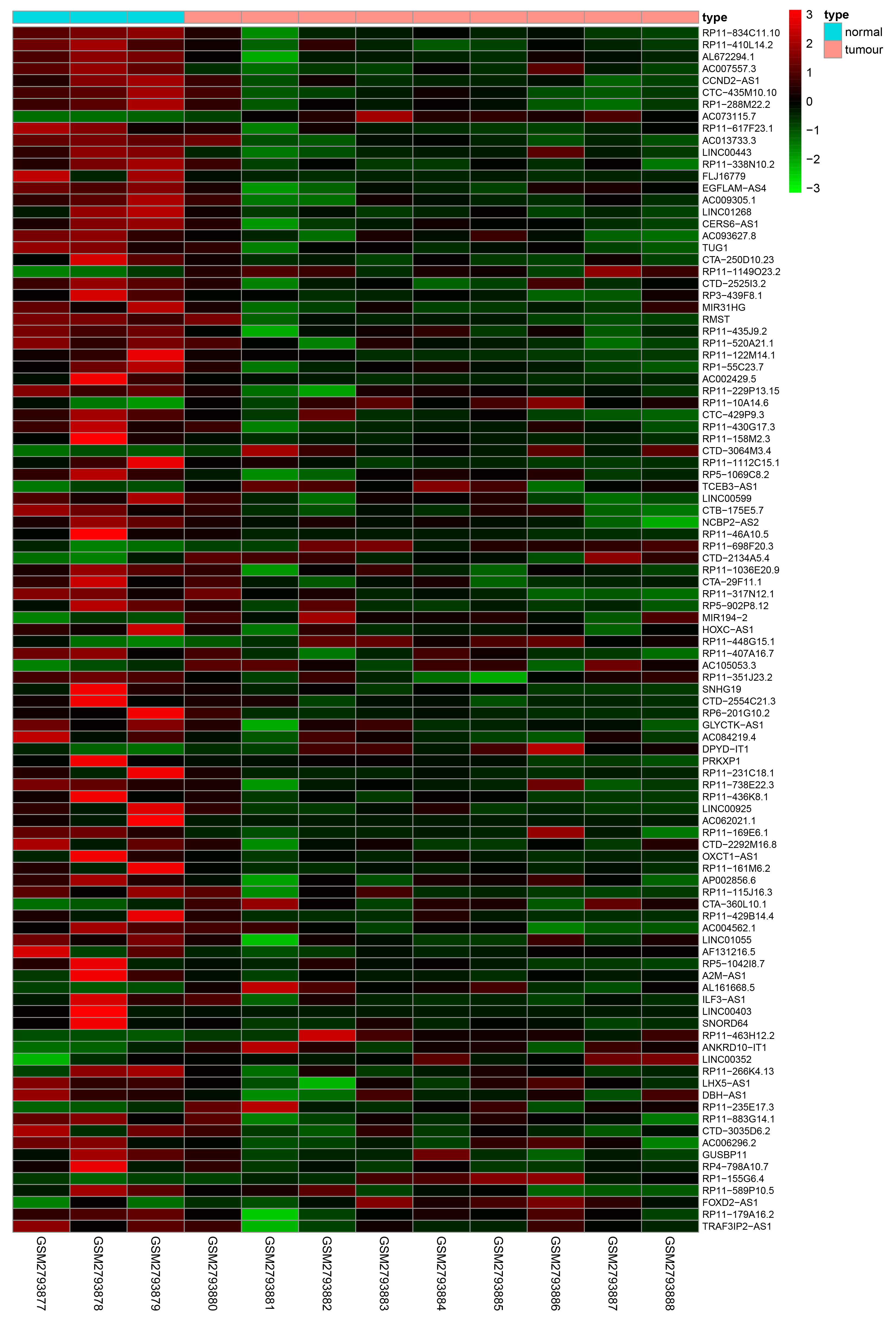

Supplement: Supplementary file 1 — Fig S1 [file JCMM-26-2620-s004.jpg]

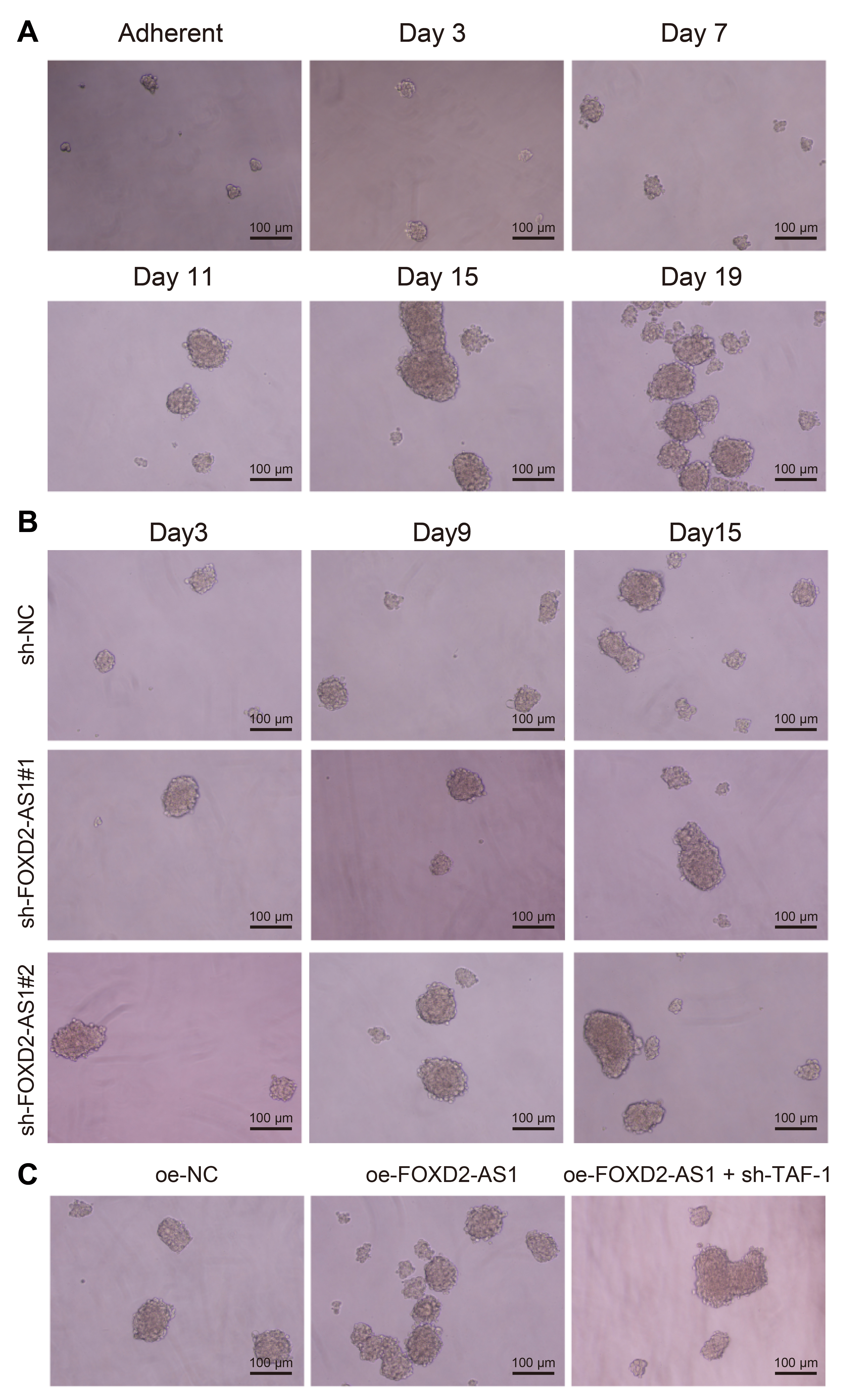

Supplement: Supplementary file 2 — Fig S2 [file JCMM-26-2620-s002.jpg]

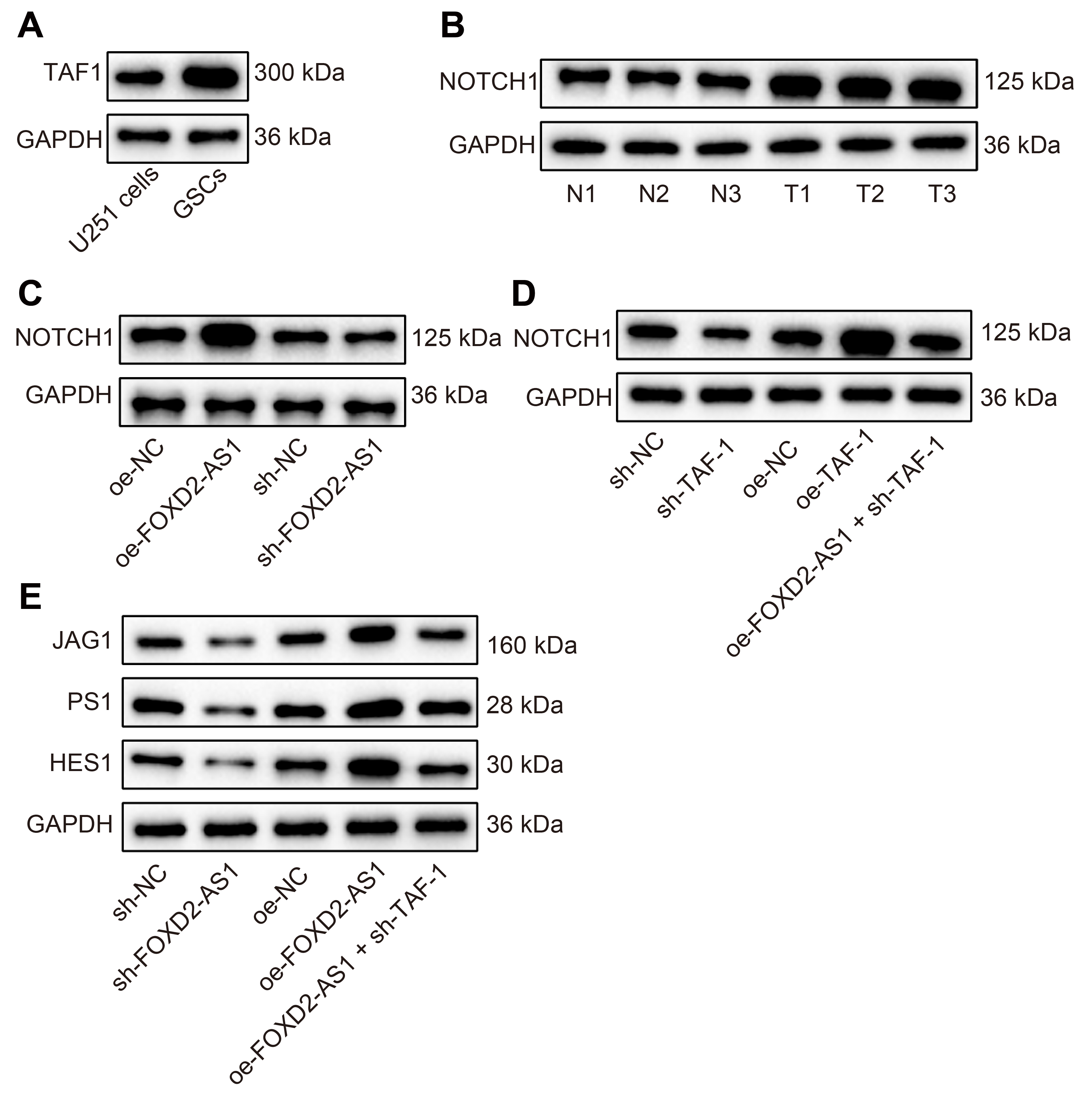

Supplement: Supplementary file 3 — Fig S3 [file JCMM-26-2620-s005.jpg]

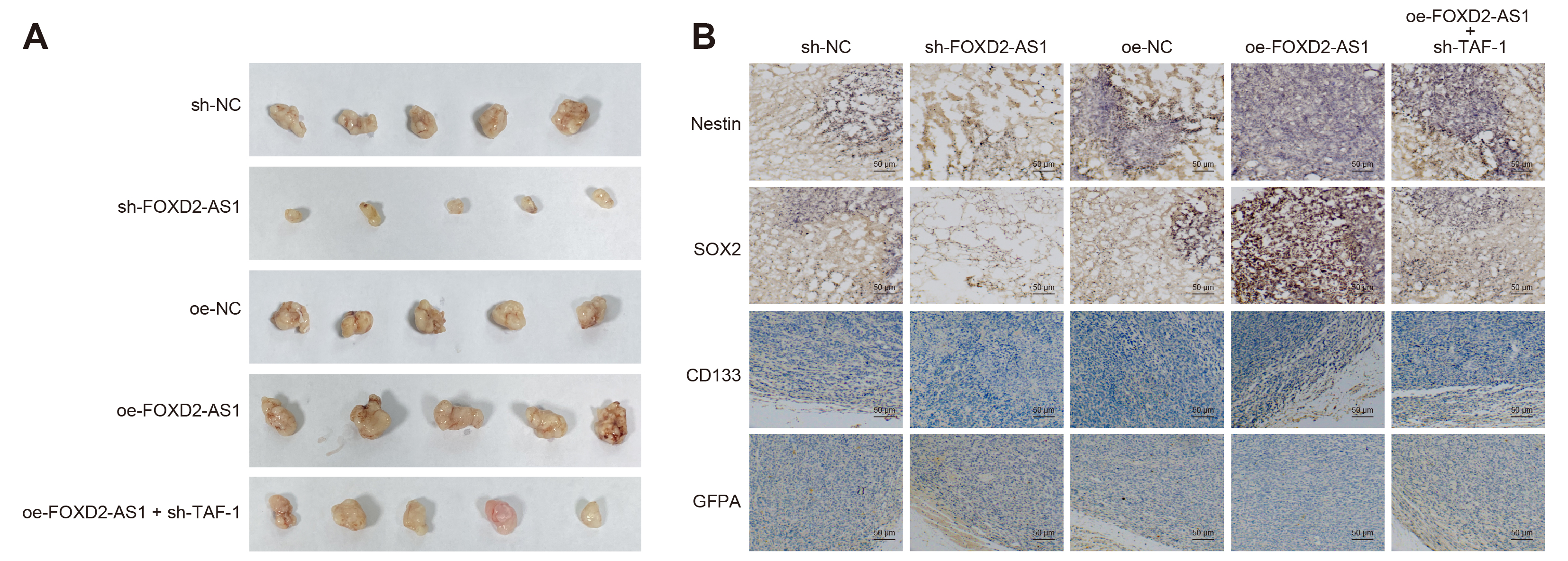

Supplement: Supplementary file 4 — Fig S4 [file JCMM-26-2620-s003.jpg]
