# Supplementary material for: Overexpression of FOXD2‐AS1 enhances proliferation and impairs differentiation of glioma stem cells by activating the NOTCH pathway via TAF‐1
Source: J Cell Mol Med. 2022 Apr 14;26(9):2620–32. doi: 10.1111/jcmm.17268 (PMC9077300; doi:10.1111/jcmm.17268)
Supplement: Supplementary file 5 — Table S1‐S2 [file JCMM-26-2620-s001.docx]

**Supplementary Table 1** Clinical characteristics

| Clinical characteristics | Case Expression NO. |
| --- | --- |
| Gender |  |
| Male | 14 |
| Female | 12 |
| Age, median (range) | 45.65 (20-71) |
| Neurological disorder |  |
| Present | 11 |
| Absent | 15 |
| Family history |  |
| Yes | 13 |
| No | 13 |
| Preoperative KPS score |  |
| ≦40 | 14 |
| >40 | 12 |
| Cigarette smoking |  |
| Ever | 9 |
| Never | 17 |
| WHO grade |  |
| I, II | 16 |
| III, IV | 10 |
| IDH |  |
| IDH-wt | 6 |
| IDH-mu | 20 |

**Supplementary Table 2** Primer sequence of qRT-PCR

|  | Primers |
| --- | --- |
| FOXD2-AS1-F | 5′-GCCCAGAACAATTGGGAGGA-3′ |
| FOXD2-AS1-R | 5′-AAGAGAGGGAGAGACGACCC-3′ |
| TAF-1-F | 5′-AACCCCTTGCTGGATGATGA-3′ |
| TAF-1-R | 5′-GGCTTAGCCTGAGGCGTGTA-3′ |
| NOTCH1-F | 5′-CTCCCCGTTCCAGCAGTCTC-3′ |
| NOTCH1-R | 5′-CAGCCACTCGCATTGACCAT-3′ |
| SOX2-F | 5′-GCCGAGTGGAAACTTTTGTCG-3′ |
| SOX2-R | 5′-GGCAGCGTGTACTTATCCTTCT-3′ |
| OCT4-F | 5′-GACAACAATGAGAACCTTCAGGAGAG-3′ |
| OCT4-R | 5′-CTGGCGCCGGTTACAGAACCA-3′ |
| Nanog-F | 5′-TCCAGCAGATGCAAGAACTCTCCA-3′ |
| Nanog-R | 5′-CACACCATTGCTATTCTTCGGCCA-3′ |
| Nestin-F | 5′-CTGCTACCCTTGAGACACCTG-3′ |
| Nestin-R | 5′-GGGCTCTGATCTCTGCATCTAC-3′ |
| CD133-F | 5′-AGAACTAGTGAGTGTTGGGTGAATCTG-3′ |
| CD133-R | 5′-GTCAAGCTTCTATCACGTAAACTAGCC-3′ |
| GAPDH-F | 5′-GCACCGTCAAGGCTGAGAAC-3′ |
| GAPDH-R | 5′-TGGTGAAGACGCCAGTGGA-3′ |
